# The New Year

**Published:** 1888-01

**Authors:** 


### HALL'S JOURNAL OF HEALTH

TRUTH DEMANDS NO SACRIFICE; ERROR CAN MAKE NONE.

VOL. 35. JANUARY, 1888. No. 1.

### THE NEW YEAR.

For the third time since the conduct of this Journal was committed to our hands, we greet our patrons with the compliments of the New Year, with an earnest desire that each and all may be able to reciprocate our good wishes with the heartiness of old and tried friends, mindful only of our efforts to impart the knowledge of those inestimable truths which sweeten the way of life and soften the pillow of the worn and weary at its close. The order of Nature is motion, agitation, progression. Science has demonstrated that all so called inanimate things, are composed of particles in a state of unceasing activity. There may be change after change in infinite variety, but in the whole universe of things there is no such thing as *death,* for out of what we term death. come new forms of life. This is an eternal law observable upon every hand. In this sense, not even the body dies. When the spirit has held it as a tenement for its appointed time, it is laid aside, having, separately considered, the same life it had before, that is to say, the elements which compose it, undergo a change, combining with different elements, thus forming other orders of life and performing anew their appointed offices in the economy of nature. What then of man, the chiefest of nature's handiwork? Is he to be reared, taught, accounted and doctored on a level with mere animals,—to live only as they live, and to die and leave no sign? Shall those God-like faculties which have equal power to estimate an atom or a world—to trace the heavenly spheres in their everlasting round of ceaseless energy, and calculate with the certainty of recurring time, the completion of a cycle of many a life-time; shall these heaven-born faculties flash upon this dull earth, only to sink into an oblivion more profound than the elements which compose man's visible entity.

It has been one of our chiefest objects in the conduct of this journal, to lay before its readers the *proofs* which are so abundant at this day, of the individual conscious existence of every soul of mortal birth in the great hereafter. We have considered the promulgation of this truth not only suited to a publication mainly devoted to health, but of the utmost consequence to those who suffer from bodily ailments oftentimes the result of mental depression, superinduced by an over-mastering dread of the unknown and misconceived future. Man should be taught to feel and know, that the present life is but the beginning of an unbroken chain of change and progression to higher levels of knowledge, which constitute the soul's growth; and of usefulness in all the ways of well doing It is to this end that we have opened our columns to those sublime evidences which show to all willing minds that the trust in a future life, which has served as the devotee's sheet-anchor in all weathers, is certain to be realized, that

> " This life of mortal breath
> Is but a suburb of the life elysian,
> Whose portal we call death."

If we have not hitherto made a sufficiently emphatic protest against the inculcation of old and worn out dogmas respecting death ; the soul's long centuries of sleep ; the far off resurrection, and the dreadful judgment, we make it now.

If our readers are ready to inquire what these things have to do with a health journal, we answer that they have everything to do with it, in preventing a morbid state of the mind almost sure to spring from either doubt or dread of the unknown and unknowable hereafter. It is indeed the acceptance of more rational views of the after life, its duties, employments and means of growth, which has transformed the King of Terrors into an Angel of Light, that ever reaches a kindly hand to the earth-worn and weary journeyer in his passage over the dark river, into the realm of light and beauty beyond.

> " Death is the crown of life :
> Were death deny'd, poor men would live in vain."

Again when the truth is made clear to the comprehension of all thinking minds, that man is a spirit now and here ; that the real seat of many of his more serious ailments is oftentimes in the mind, as distinguished from the body and that the true remedy is to be found in words of cheer and deeds of kindness, that more than all else

> "Minister to a mind diseased"

rather than in irritating drugs, the physician of the future will approach his patient with a better understanding of what is needful to restore his dormant energies to healthy action, or if in natural course the time has come for the spirit to lay aside its earthly tenement, he may be permitted to depart in peace, untortured by useless attempts to stay the hand of time, and undismayed by the dread of being brought to judgment before an angry, revengeful God, and given over to the endless torture of His insolent rival. In a word the art of healing will reach the *cause* of disease, and the terrible dread of death which haunts the parting shore like a spectre, will give place to visions of beauty wherein the forms of loved ones gone before, wait with outstretched arms to conduct the liberated spirit to the abode which it has earned for itself by good deeds done in the body.

> "He most lives
> Who thinks most, feels the noblest, acts the best."

